# Role of Apoptotic Proteins in REC-2006 Mediated Radiation Protection in Hepatoma Cell Lines

**DOI:** 10.1093/ecam/neq059

**Published:** 2011-03-20

**Authors:** Pankaj Kumar Singh, Raj Kumar, Ashok Sharma, Rajesh Arora, Raman Chawla, Swatantra Kumar Jain, Rajendra Prasad Tripathi, Rakesh Kumar Sharma

**Affiliations:** ^1^Institute of Nuclear Medicine and Allied Sciences, Delhi, India; ^2^Jamia Hamdard, Hamdard Nagar, Delhi, India

## Abstract

The present study was carried out to evaluate the role of apoptotic proteins in REC-2006-mediated radiation protection in hepatoma cell lines. REC-2006 treatment 2 h before irradiation strongly inhibited the cleavage of ATM and PARP-1 in HepG2 cells. The expression of nuclear apoptosis inducing factor (AIF) was found to be more inhibited (~17%) in HepG2 cells in REC-2006 + radiation-treated group. More inhibition (*~*33%) of cytochrome *c* was observed in HepG2 cells upon REC-2006 treatment 2 h prior irradiation. Similarly, significantly more (*P<.05*) inhibition of Apaf-1, caspase-9 and caspase-3 was observed in REC-2006 + radition-treated group in HepG2 cells. REC-2006 treatment restored the expression of ICAD in HepG2 cells; however, no restoration was observed in Hep3B cells. Lower nuclear to cytoplasmic CAD ratio was observed in HepG2 cells (~0.6) as compared with Hep3B cells (~1.2) in REC-2006 + radiation-treated group. In conclusion, REC-2006 rendered higher protection in HepG2 cells by inhibiting the expression and translocation of AIF, inhibiting the cleavage of ATM and PARP-1, restoring the expression of ICAD, inhibiting the release of cytochrome *c* and thus modulating the expression of Apaf-1 caspase-9 and activity of caspase-3.

## 1. Introduction

Exposure to ionizing radiation (IR) resulted in complex physiological responses in the biological systems [1–3]. IR is known to induce apoptosis [4, 5]. Damage to DNA is considered as a major initiator of cellular response to IR [6]. The p53 gene is activated in response to DNA damage and encodes a transcription factor [7], which in turn activates genes that arrest cell growth and/or induce apoptosis, thereby preventing the propagation of genetically damaged cells. In the absence of cellular stress, p53 protein is maintained at low steady-state levels and exerts very little, if any, effect on the fate of the cell. However, in response to various types of cellular stress, p53 protein is activated and this is reflected in elevated protein levels, as well as augmented biochemical capabilities. As a consequence of p53 activation, cells can undergo marked phenotypic changes, ranging from increased DNA repair to senescence and apoptosis [8].

The activation of the cysteine proteases with aspartate specificity, termed caspases, is an extremely important step in the execution of programmed cell death [9]. These proteases are highly specific in their action and activate or inhibit a variety of key protein molecules in the cell. Strictly defined, cell death can only be classified to follow the classical apoptotic mode if execution of cell death is dependent on caspase activity [9]. There are two relatively well-characterized caspase cascades. One is initiated by the activation of cell surface receptors, such as Fas and tissue necrosis factors, leading to caspase-8 activation, which in turn cleaves and activates downstream caspases such as caspase-3, -6 and -7 [10]; and the other is triggered by cytochrome *c* released from mitochondria, which promotes the activation of caspase-9 through apoptosis protease activating factor-1 (Apaf-1) [11]. Apart from caspase-mediated induction of apoptotic cell death, apoptosis inducing factor (AIF) has also been shown as a main actor behind the caspase-independent death pathway [12].

During apoptosis, the activated caspases are known to cleave substrates such as Poly (ADP-ribose) polymerase (PARP-1), actin, fodrin and lamin [13]. Caspase-3 has been shown to cleave inhibitor of caspase-activated DNase (ICAD) to inactivate its caspase-activated DNase (CAD)-inhibitory effect [13].

Ataxia Telanagiectasia Mutated (ATM) and PARP-1 are two of the most important players in the cell's response to DNA damage. PARP-1 and ATM recognize and bind to both single- and double-strand DNA breaks in response to different triggers [14]. The cleavage of ATM during apoptosis abrogates its protein kinase activity against p53 and generates a kinase-inactive protein that acts through its DNA-binding ability in a *trans*-dominant-negative fashion to prevent DNA repair [15]. The ATM protein kinase is centrally involved in the cellular response to IR and other DNA double-strand-break-inducing insults.

PARP-1 is a nuclear enzyme, which is activated in response to genotoxic insults by binding damaged DNA and attaching polymers of ADP-ribose to nuclear proteins at the expense of its substrate NAD^+^ [14]. The proteins respond to DNA damage by transferring 50–200 molecules of ADP-ribose to various nuclear proteins, including transcription factors, histones and PARP-1 itself [16]. This poly(ADP-ribosyl)ation activity of PARP-1 is important for maintaining genomic integrity [17] and it has been associated with longevity. Furthermore, PARP-1 is activated by agents infringing single-stranded DNA damage such as alkylating agents, IR and oxidative damage. Diverse apoptotic signals for caspase activation converge at the mitochondria level, provoking the release of cytochrome *c*, which participates in the central control or executioner phase of the cell death cascade [18, 19].

The release of cytochrome *c* triggers the formation of a complex containing Apaf-1, a mammalian CED-4 homologue and procaspase-9, which is then auto-processed and thereby capable of processing downstream effector procaspases such as procaspase-3 [11]. The processing of these caspases is followed by the cleavage of apoptotic substrates, leading to the disruption of important cellular processes, changes in cellular and nuclear morphology and ultimately cell death [20].

Previously, we reported higher radioresistance against *γ*-radiation in the HepG2 (p53^++^) cell lines as compared with the Hep3B (p53^−−^) cell lines, indicating the plausible role of p53 in radioresistance [2]. We demonstrated that a non-polar chloroform fraction (REC-2006) of *Podophyllum hexandrum*, a Himalayan plant which grows at an altitude of about 4000 m in the Himalayan region, rendered nearly twice the survival (dose modification factor (DMF) = 1.72) in p53 expressing HepG2 cells, as compared with p53-negative Hep3B cells (40% survival; DMF = 1.26) [2], mainly by modulating the expression of p53. REC-2006 persuaded cell-cycle arrest in the G1 phase, encouraged cell proliferation and DNA repair and thus rendered significantly higher protection in the HepG2 cell line against acute *γ*-radiation. Conversely, in the absence of p53, damaged DNA incorporated into the next generation leading to cell death in the Hep3B cell line [2]. We also reported a potent p53 inhibitor (pifithrin-*α*), debilitated the radioprotective potential of REC-2006 in the HepG2 cell line indicating the role of p53 protein in REC-2006 mediated radioprotection [3]. To determine the effect of *γ*-radiation on HepG2 and Hep3B cells, colony formation assay was used and ∼80% (*P* < .05) mortality was observed in HepG2 (p53^++^) cells at 10 Gy (LD_80_ = 10 Gy). Whereas, a similar level (∼80%) of lethality was observed in Hep3B (p53^−−^) cells at 3.7 Gy only (LD_80_ = 3.7 Gy). This was used as study model. Previously we have reported that the most optimal (maximum) survival (80%) was observed in HepG2 (p53^++^) cell line upon REC-2006 (10^−5^ *μ*g mL^−1^) treatment 2 h before irradiation (10 Gy) (LD_80_ = 10 Gy). However, only 40% survival (maximum) was achieved at the same drug concentration (10^−5^ *μ*g mL^−1^) at 3.7 Gy (LD_80_ = 3.7 Gy) in the Hep3B cell line. With a view to further elucidating the mode of action at molecular level, the present study was carried out to investigate the expression pattern of apoptosis regulatory proteins in REC-2006/*γ*-radiation-treated groups in HepG2 and Hep3B cell lines with a view to decipher the precise role of apoptotic proteins in REC-2006-mediated radiation protection.

## 2. Methods

### 2.1. Reagents

The reagents used were of analytical grade and standard make. Culture medium, minimal essential medium (MEM), antibiotics (penicillin G and streptomycin), trypsin and fetal bovine serum (FBS) were procured from HiMedia, India and Sigma Aldrich, USA. Mouse monoclonal antibodies for the detection of human p53, ATM, PARP-1, AIF, cytochrome *c*, caspase-3, caspase-9, ICAD, CAD and *β*-actin proteins and alkaline phosphate-conjugated secondary anti-mouse antibodies were procured from Santa Cruz Biotech (Santa Cruz, CA, USA). Dithiothreitol (DTT), ethylene diamine tetra-acetic acid (EDTA), PMSF, Nonidet P40 (NP-40), 5-bromo-4-chloroindol-3-yl phosphate/Nitro Blue Tetrazolium (BCIP-NBT) reagent and protease inhibitors were procured from Sigma Aldrich, St Louis, MO, USA. Lysine, Tris-base and sodium dodecyl sulfate (SDS) were obtained from Merck, Darmstadt, Germany, whereas nitrocellulose membrane was purchased from Millipore, USA.

### 2.2. Plant Extract

REC-2006 fraction was extracted from rhizomes of Indian *P. hexandrum* as described earlier [1, 2]. Dried rhizomes of *P. hexandrum* Roylle growing at an altitude of 4000 m in the Himalayan region were procured from the Defence Institute of High Altitude Research (DIHAR), formerly Field Research Laboratory (Leh, Jammu and Kashmir, India). Dried powder (10 g per 100 mL, w/v) of *P. hexandrum* rhizome was extracted in a Soxhlet apparatus thrice with different solvents (1 : 6 ratio) of increasing polarity namely, hexane, chloroform, alcohol, 50% alcohol in water and water subsequently over the course of 24–72 h. The respective filtrates were combined. All the extracts were filtered through Whatman filter paper no. 3, followed by filtration through a 0.22 *μ*m filter (Millipore, USA). Extracts were than concentrated by solvent evaporation under reduced pressure in a rotary evaporator (Buchi, Flawil, Switzerland) and dried. Out of the five different fractionated extracts prepared, the dried chloroform extract (code name: REC-2006) was used for the present study [2, 3].

### 2.3. Cell Culture

The cell culture studies were performed on human hepatoma cell lines. HepG2 (p53^++^, carrying wild-type p53) and Hep3B (p53^−−^ carrying p53 null) cells were purchased from the National Centre for Cell Science (NCCS), Pune, India. The cells were maintained as monolayer culture in MEM (supplemented with 10% FBS, 100 U mL^−1^ of penicillin, 100 *μ*g mL^−1^ of streptomycin, pH 7.4) at 37°C in a humidified 5% CO_2_ incubator (Binder, Germany) in 25-cm^2^ culture flasks (Nunc USA.) and were sub-cultured twice a week. All experiments were performed done when the cells reached about 70% confluency.

### 2.4. Harvesting of Adherent Cells

Cells were harvested using 0.25% trypsin in Hank's balanced salt solution (HBSS). After removal of the medium, 1.0 mL of chilled trypsin (0.25%) was poured into the culture flask (T25) and left for 30 s at room temperature. Excess trypsin, was decanted and the culture flasks were incubated at 37°C till the cells started rolling off. Harvested cells were counted and proper dilutions were made by adding complete MEM medium to the cell suspension for further experimentation.

### 2.5. Irradiation

Radiation was delivered using a ^60^Co gamma chamber (Model 220, Atomic Energy of Canada Ltd.) (dose rate of 43.8 cGy/min). Cells were cultured in culture flasks (25 cm^2^) and irradiated at their respective LD_80_ radiation doses (HepG2: LD_80_ = 10 Gy; Hep3B: LD_80_ = 3.7 Gy). Culture flasks fitted with a filter cap (Nunc, USA) were used to avoid the generation of hypoxic conditions in the irradiation chamber [2].

### 2.6. Colonogenic Assay for Cell Survival

Cells (HepG2 and Hep3B) harvested from exponentially grown cultures and counted. Viability of cells was tested by dye exclusion test (0.1% Trypan Blue). Nearly 300 cells were seeded in 60-mm culture dishes (Tarsons, India) and incubated for 12 h. The attached cells were then treated with REC-2006, *γ*-radiation and REC-2006 2 h before *γ*-radiation. After treatments, the cells were further incubated at 37°C with 5% CO_2_ in a humidified condition. Colonies appeared nearly 2 weeks of incubation after and were counted. All experiments were carried out in triplicate.

### 2.7. Experimental Plan

The experiment was divided into the following four groups. Each group was in triplicates.

1. Group I. Untreated control of HepG2 and Hep3B cells.
2. Group II. HepG2 and Hep3B cells treated with 10^−5^ *μ*g/mL) of REC-2006.
3. Group III. HepG2 and Hep3B cells exposed to their respective acute irradiation (HepG2: LD_80_ = 10 Gy; Hep3B: LD_80_ = 3.7 Gy) [2].
4. Group IV. HepG2 and Hep3B cells exposed to radiation (respective LD_80_) 2 h after REC-2006 (10^−5^ *μ*g mL) treatment.

HepG2 and Hep3B cells were divided into the above mentioned four treatment groups and after final treatment, cells were harvested at 8 h for protein expression studies.

### 2.8. Extraction of Cytoplasmic and Nuclear Protein Fraction

Cytoplasmic and nuclear protein fraction was extracted as described earlier [2]. HepG2 and Hep3B cells were harvested by trypsinization and centrifuged at 100 g for 5 min. The pellet was resuspended in 300 *μ*L of buffer A (50 mM NaCl, 10 mM HEPES (4-(2-hydroxyethyl)-1-piperazineethanesulfonic acid) pH 8.0, 500 mM sucrose, 1 mM EDTA, 0.5 mM spermidine, 0.15 mM spermine, 0.2% Triton X-100) containing *β*-mercaptoethanol and the protease inhibitors, that is, Phenylmethanesulfonyl fluoride (PMSF), leupeptin, aprotinin and pepstatin. After 15 min of incubation on ice, the suspension was centrifuged at 1400 g for 10 min. The supernatant (cytoplasmic fraction) was collected and stored, whereas the pellet was washed with 200 *μ*L buffer B (50 mM NaCl, 10 mM HEPES pH 8, 25% glycerol, 0.1 mM EDTA, 0.5 mM spermidine, 0.15 mM spermine) and then resuspended in 100 *μ*L buffer C (350 mM NaCl, 10 mM HEPES, 25% glycerol, 0.1 mM EDTA, 0.5 mM spermidine, 0.15 mM spermine). After centrifugation at 17 000 g the supernatant (nuclear fraction) was collected and stored at 4°C [2].

### 2.9. Protein Estimation

Total soluble protein content in the different nuclear and cytoplasmic fractions were estimated as described earlier [2, 21]. Briefly, 10 *μ*L of the sample was mixed with 90 *μ*L distilled water. After mixing thoroughly, Bradford reagent (1.0 mL) was added and absorbance was recorded at room temperature within 20–30 min at 595 nm using a micro-titer plate reader (Wallac, USA). The amount (micrograms) of protein was quantified using BSA standard curve.

### 2.10. SDS-PAGE Analysis of Proteins

For SDS-PAGE analysis, 10% polyacrylamide gels of 0.75 mm thickness were prepared, whereas for analysis of ATM and PARP-1, 7% polyacrylamide gels of 0.75 mm thickness were used. Protein fractions were added to SDS-PAGE sample buffer (0.0625 M Tris-HCl, pH 6.8; 2% (w/v) SDS; 5% (v/v) glycerol; 2% (v/v) 3-mercaptoethanol; 0.01% (w/v) bromophenol blue) and heated in a boiling water bath for 2-3 min. Equal amount of protein samples (10 *μ*g) were loaded in each well. Electrophoresis was carried out at a constant voltage (stacking at 60 V, resolving at 70 V). After electrophoresis, the gels were stained with gentle shaking in 0.1% coomassie brilliant blue R-250 in methanol: glacial acetic acid: water (4 : 2 : 4, v/v/v) at room temperature and destained in a washing solution (methanol: acetic acid: water (1 : 0.7 : 8.3)) to obtain distinct bands over clear background. The gels were stored in 0.1% acetic acid for future analysis [2, 22].

### 2.11. Western Blot Analysis

Proteins were transferred electrophoretically (100 V, 1 h) onto a nitrocellulose membrane using a mini-trans blot assembly (Bio-Rad, Hercules, CA, USA). The membrane was blocked in a blocking solution (containing 5% w/v skimmed milk in TBS) for 2 h at room temperature. The expression levels of p53, ATM, PARP-1, AIF, cytochrome *c*, caspase-3, caspase-9, ICAD, CAD and *β*-actin were analyzed by probing with respective mouse monoclonal antibodies (1 : 1000 dilution) having cross reactivity with human proteins. Three washes of 15 min each in washing buffer (TBS, 0.2% Tween 20), were grown and the membranes were incubated in TBS containing goat anti-mouse IgG alkaline phosphate conjugated secondary antibodies (1 : 10 000 dilution). The membranes were again washed (three times for 15 min) each with washing buffer and then treated with BCIP-NBT reagent (Sigma, St Louis, MO, USA) for 10–30 min [23] followed by densitometric analysis. The quantification of individual protein bands was done using Alpha Ease FC 4.0.0 Software (Alpha Innotech, India). Relative expression of proteins was evaluated by normalizing the expression of proteins with quantitative housekeeping protein *β*-actin.

### 2.12. Statistical Analysis

The data is presented as mean ± SD of three separate experiments each performed in triplicate. The statistical two-way analysis of variance (ANOVA) was performed for multiple comparisons, followed by post hoc test. Effect of different concentrations on survival of cell lines was analyzed using Dunnet's *t*-test with two controls and five test values. Correlation analysis was performed to evaluate various dose-dependent effects. All the statistical tests were performed using statistical software SPSS Version 11. Significance was tested and *P* < .05 was considered as level of significance.

## 3. Results

### 3.1. p53 and ATM Expression Analysis

**Figure 1:**
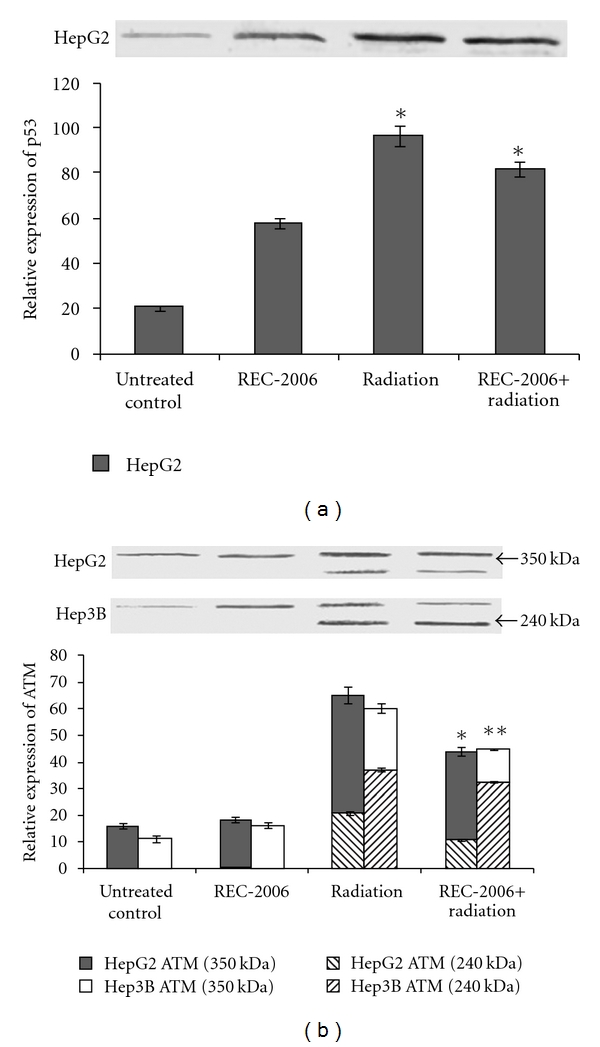
Effect of optimal concentration of drug (REC-2006), that is, 10^−5^ *μ*g mL^−1^ given 2 h prior to irradiation (LD_80_ of respective cell lines) on expression of (a) p53; (b) ATM in HepG2 and Hep3B cell lines. Single asterisk indicates significant radiomodulatory activity (increase/decrease in expression) in HepG2 cell lines, whereas double asterisks indicate the corresponding effect in Hep3B cell lines with respect to radiation (LD_80_) controls.

A significant (*P* < .05) increase in the expression of p53 was observed in HepG2 (p53^++^) cells treated with REC-2006 alone (by 30 ± 3.7%) and irradiation (by 73 ± 4.2%) as compared to untreated control. However, REC-2006 treatment 2 h before irradiation decreased (16 ± 2.75%) the expression of p53 as compared with only irradiated group of HepG2 cell line (Figure 1(a), lanes 3 and 4). As predicted, no expression of p53 was observed in any treatment group of Hep3B (p53^−−^) cell line (Figure 1(a)).

The expression of ATM (both fragment) increased in radiation (+/− REC-2006) treatment group as compared to untreated control of HepG2 (62%) as well as Hep3B (67%) cell line. Treatment of *γ*-radiation produced more cleavage product (249-kDa fragment) in Hep3B cell line as compared with the HepG2 cell line (Figure 1(b), lane 3). However, in a REC-2006 + radiation-treated group, more inhibition of ATM cleavage was observed in HepG2 cells, as compared with Hep3B cells (Figure 1(b), lanes 3 and 4).

### 3.2. Cytoplasmic and Nuclear PARP-1 Expression Analysis

**Figure 2:**
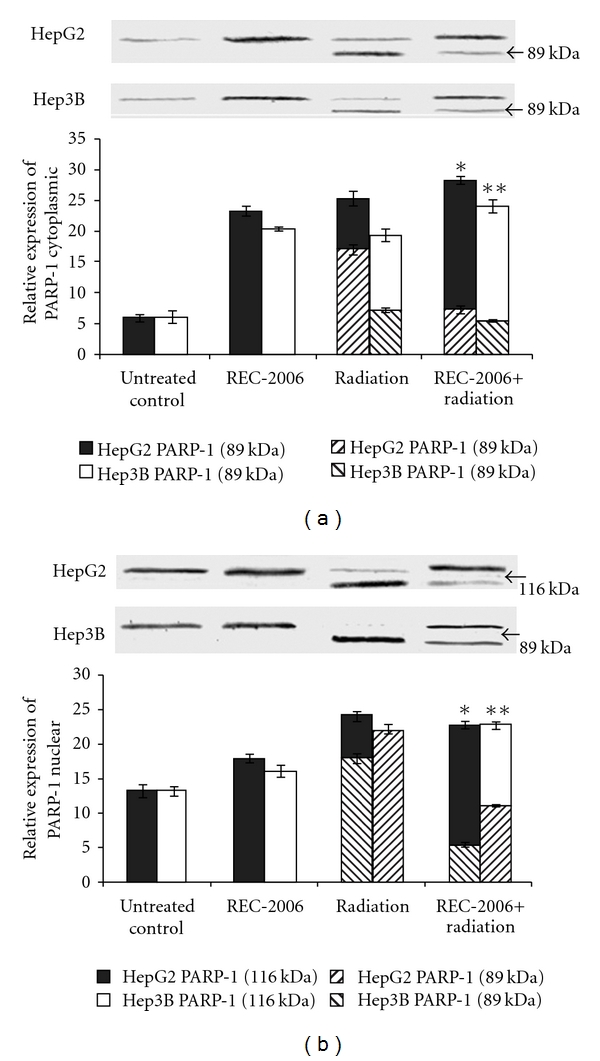
Effect of optimal concentration of drug (REC-2006), that is, 10^−5^ *μ*g mL^−1^ given 2 h prior to irradiation (LD_80_ of respective cell lines) on expression of (a) cytoplasmic PARP-1; (b) nuclear PARP-1 in HepG2 and Hep3B cell lines. Single asterisk indicates significant radiomodulatory activity (increase/decrease in expression) in HepG2 cell lines, whereas double asterisk indicate the corresponding effect in Hep3B cell lines with respect to radiation (LD_80_) controls.

To determine the effect of REC-2006 treatment on the modulation of PARP-1 expression, in cytoplasm as well in the nucleus, the cytoplasmic and nuclear proteins was separated and analyzed independently. REC-2006 treatment alone increased the expression of cytoplasmic PARP-1 in HepG2 (∼23%) and Hep3B (∼20%) cells, as compared to the respective untreated control (Figure 2(a), lanes 1 and 2). Caspase-mediated cleavage of cytoplasmic PARP-1 was observed in both cell lines in the radiation-treated groups. More inhibition in nuclear translocation of fragmented PARP-1 (89 kDa) was observed in HepG2 cell line, as compared with Hep3B cell line when REC-2006 treatment was given 2 h before irradiation. Inhibition of caspase-mediated cleavage of PARP-1 (89 kDa) was observed when REC-2006 was treated 2 h before irradiation in HepG2 and Hep3B cells. In radiation (−2 h REC-2006) treatment group, intact (116 kDa) to cleaved (89 kDa) PARP-1 ratio was observed to be much higher in nuclear fraction of HepG2 cells (∼3.3) as compared with Hep3B cells (∼0.91) (Figure 2(b), lane 4).

### 3.3. Cytoplasmic and Nuclear AIF Expression Analysis

**Figure 3:**
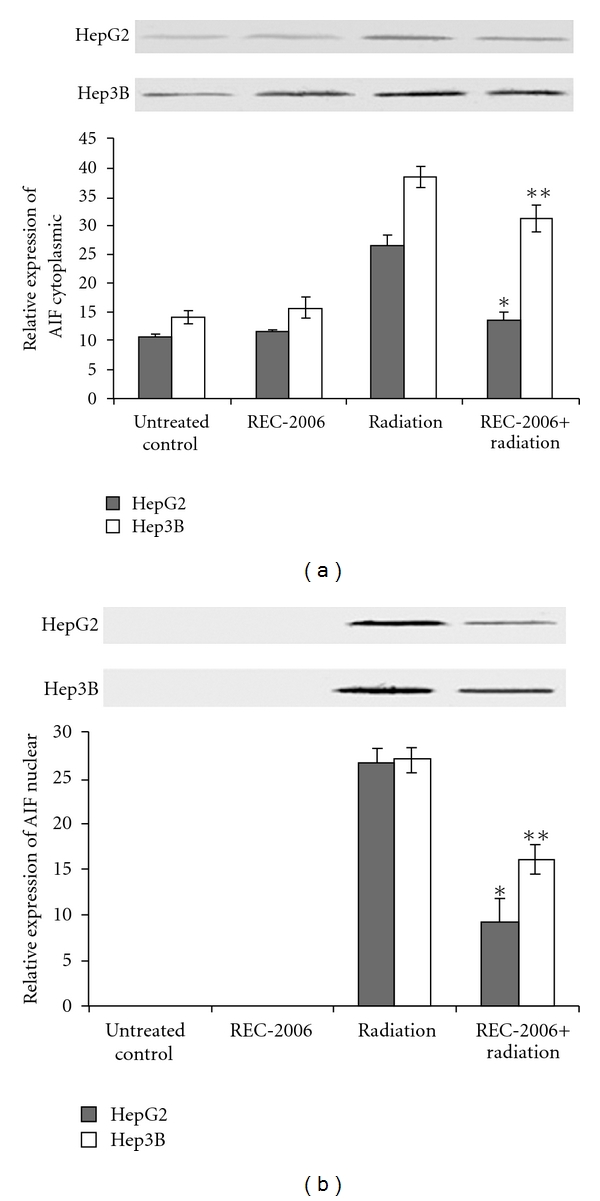
Effect of optimal concentration of drug (REC-2006), that is, 10^−5^ *μ*g mL^−1^ given 2 h prior to irradiation (LD_80_ of respective cell lines) on expression of (a) cytoplasmic AIF; (b) nuclear AIF in HepG2 and Hep3B cell lines. Single asterisk indicates significant radiomodulatory activity (increase/decrease in expression) in HepG2 cell lines, whereas double asterisks indicate the corresponding effect in Hep3B cell lines with respect to radiation (LD_80_) controls.

REC-2006 treatment alone rendered no considerable effect on the expression of cytoplasmic AIF HepG2 and Hep3B cell lines, when compared with their respective untreated control groups (Figure 3(a), lanes 1 and 2). No expression of nuclear AIF was observed in untreated and REC-2006 treated HepG2 and Hep3B cell lines (Figure 3(b), lanes 1 and 2). Expression of cytoplasmic AIF was strongly inhibited (∼50% decrease) in HepG2 (p53^++^) cells pretreated with REC-2006 2 h before irradiation as compared with irradiated control (Figure 3(a), lanes 3 and 4). However, the degree of inhibition was relatively less (∼10% decrease) in the respective treatment group of Hep3B cell line (Figure 3(a), lanes 3 and 4). Similarly, the expression of nuclear AIF was found to be inhibited in HepG2 cell line (∼17%), as compared with Hep3B cell line (∼11%) in REC-2006 + radiation-treated group, and also when compared with their respective radiation-only treatment group (Figure 3(b), lanes 3 and 4).

### 3.4. Modulation of Cytochrome *c*, Apaf-1, Caspase-9 and Caspase-3

**Figure 4:**
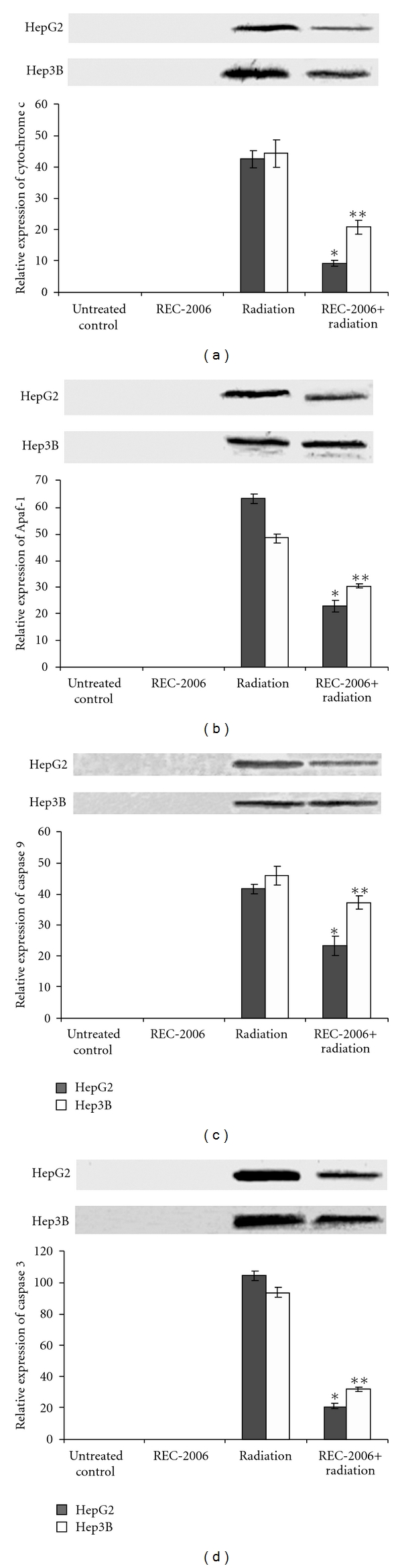
Effect of optimal concentration of drug (REC-2006), that is, 10^−5^ *μ*g mL^−1^ given 2 h prior to irradiation (LD_80_ of respective cell lines) on expression of (a) cytochrome *c*; (b) Apaf-1; (c) caspase-9; (d) caspase-3 in HepG2 and Hep3B cell lines. Single asterisk indicates significant radiomodulatory activity (increase/decrease in expression) in HepG2 cell lines, whereas double asterisks indicate the corresponding effect in Hep3B cell lines with respect to radiation (LD_80_) controls.

A decrease in the expression of cytochrome *c* was observed in REC-2006 pre-treated (−2 h) irradiated group as compared with only irradiated group of both HepG2 and Hep3B cells. However, higher inhibition of cytochrome *c* was observed in HepG2 cells (∼33% decrease) as compared with Hep3B cells (∼24% decrease) upon REC-2006 treatment 2 h prior irradiation (Figure 4(a), lanes 3 and 4). No expression of cytochrome *c* was observed in REC-2006 treated and untreated control group of HepG2 and Hep3B cells (Figure 4(a), lanes 1 and 2). Similarly higher inhibition of Apaf-1 was observed in HepG2 cells (∼40% decrease) as compared with Hep3B cells (∼19% decrease) upon REC-2006 treatment 2 h prior irradiation (Figure 4(b), lanes 3 and 4). However, no expression of Apaf-1 was observed in untreated control and REC-2006-treated group of HepG2 as well as Hep3B cells (Figure 4(b), lanes 3 and 4). Similarly, no expression of caspase-9 was observed in untreated control and REC-2006-treated group of HepG2 as well as Hep3B cells. A substantial decrease in caspase-9 expression was observed in HepG2 cells (∼14% decrease) as compared with Hep3B cells (∼8% decrease) when REC-2006 treatment was given 2 h prior irradiation (Figure 4(c), lanes 3 and 4). The expression of caspase-3 decreased upon REC-2006 (−2 h) + radiation-treated group as compared with irradiated group in both HepG2 and Hep3B cell lines. However, higher inhibition of caspase-3 was observed in HepG2 cells (*∼*84% decrease) as compared with Hep3B cells (∼62% decrease) upon REC-2006 treatment 2 h prior to irradiation (Figure 4(d), lanes 3 and 4). No expression of caspase-3 was observed in REC-2006-treated and untreated control group of HepG2 and Hep3B cells (Figure 4(d), lanes 1 and 2).

### 3.5. Modulation of Cytoplasmic ICAD and Cytoplasmic and Nuclear CAD

**Figure 5:**
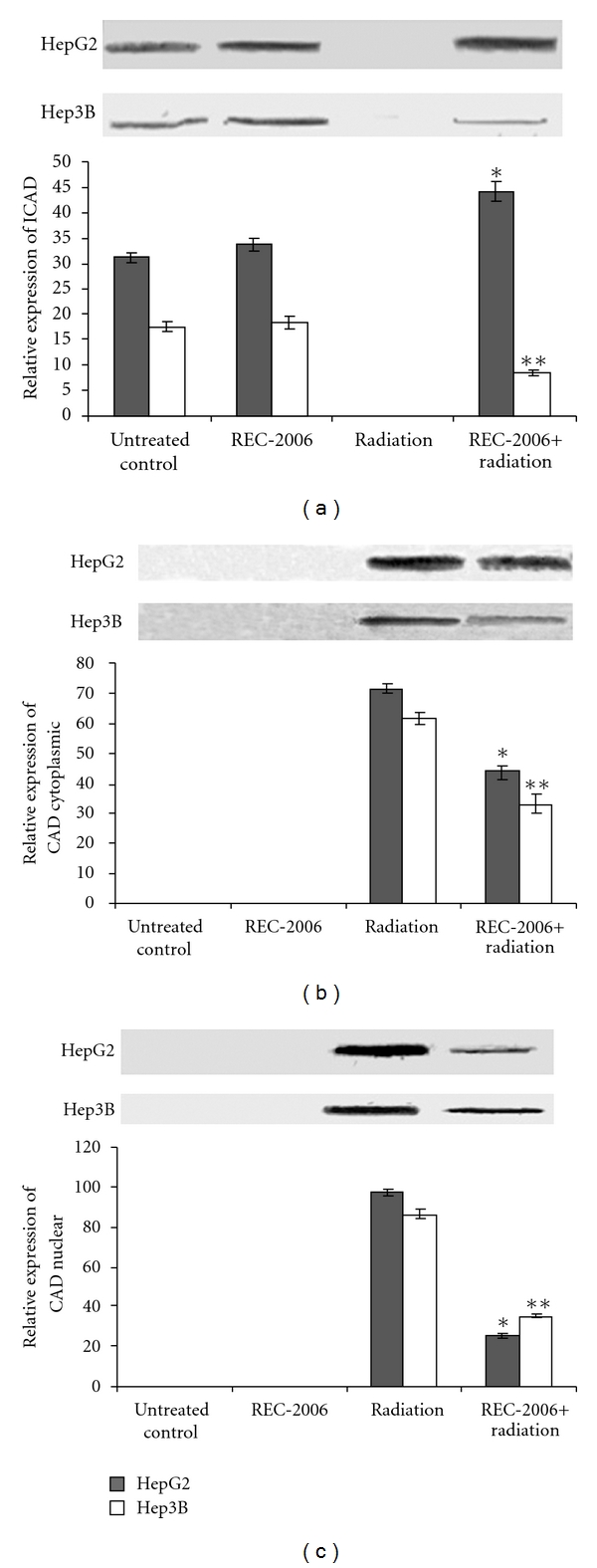
Effect of optimal concentration of drug (REC-2006), that is, 10^−5^ *μ*g mL^−1^ given 2 h prior to irradiation (LD_80_ of respective cell lines) on expression of (a) ICAD; (b) cytoplasmic CAD; (c) nuclear CAD in HepG2 and Hep3B cell lines. Asterisk indicates significant radiomodulatory activity (increase/decrease in expression) in HepG2 cell lines, whereas double asterisks indicate the corresponding effect in Hep3B cell lines with respect to radiation (LD_80_) controls.

The expression of cytoplasmic ICAD was found to be inhibited in radiation-treated cancers group as compared with untreated control in HepG2 as well as Hep3B cells. However, REC-2006 treatment 2 h prior irradiation significantly (*P* < .05) restored the expression of ICAD in HepG2 cells. However, no REC-2006-mediated restoration of ICAD was observed in Hep3B (Figure 5(a), lane 4). An inhibition in the expression of cytoplasmic as well as nuclear CAD was observed in HepG2 as well as Hep3B cells treated with REC-2006 (−2 h) + radiation as compared with only irradiated control groups (Figures 5(b) and 5(c), lanes 3 and 4). In a REC-2006 (−2 h) + irradiation treatment group, lower nuclear to cytoplasmic CAD ratio was observed in HepG2 cells (∼0.6) as compared with Hep3B cells (∼1.2). Lower nuclear to cytoplasmic CAD ratio indicated the REC-2006-mediated inhibition of nuclear translocation of CAD in HepG2 cells (Figures 5(b) and 5(c), lane 4).

## 4. Discussion

During the course of evolution, cells have evolved various sophisticated pathways to sense and overcome DNA damage as a mechanism to preserve the integrity of the genome. Radiation and/or environmental toxins induce spontaneous DNA lesions, trigger checkpoint activation and consequent cell-cycle arrest leading to DNA repair or apoptosis. Exposure to IR results in the activation of complex signal transduction pathways, which determine response of cells and organisms. Differences in cell type, interindividual genetic differences and crosstalk occurring between signaling pathways help to channelize radiation stress signals between cell-cycle delay, enhanced DNA repair and apoptosis [6]. The discovery of the p53 tumor suppressor gene initiated a plethora of investigation primarily to understand the basic biology behind its role in maintaining genomic stability and cellular response to DNA damage. p53 is one of the most commonly mutated genes in human cancers [6] and its product is a multifunctional protein that regulates several physiological processes including cell-cycle checkpoints, apoptosis and DNA repair [24].

*Podophyllum hexandrum* Royle, a high altitude Himalayan medicinal plant is being used since ancient times in the Indian and Chinese systems of medicine for the treatment of various diseases and disorders and its radioprotective properties of *P. hexandrum* have been established in our institute recently [2, 3, 5, 21–23, 25–32]. REC-2006 contains 4-demethylpodophyllotoxin, podophyllotoxin glycoside, epi-podophyllotoxin and podophyllotoxin [29]. It has been reported earlier that aryl-tetralin lignan content influences the radiation protective potential of the Podophyllum fractions to a great extent [30].

We earlier reported that REC-2006 modulated the expression of p53 and thereby persuaded cell-cycle arrest in the G1 phase, encouraged cell proliferation and DNA repair and thus rendered significantly higher protection in the HepG2 cell line against acute *γ*-radiation. Conversely, Singh and co-workers reported the LD_80_ (80% death) of HepG2 cell at the dose of 10 Gy (dose rate of 43.8 cGy min^−1^) [2]. In order to simulate the same effect of radiation in Hep3B cells, we took LD_80_ of Hep3B cell (3.7 Gy: dose rate 43.8 cGy min^−1^) as a radiation control for Hep3B cells. It has been reported that in the absence of p53, damaged DNA was incorporated into the next generation leading to cell death in the Hep3B cell line [2]. The present study was carried out in continuation to investigate the role of apoptotic proteins in REC-2006 mediated radiation protection.

A significant increase in the expression of p53 was observed in the HepG2 (p53^++^) cells treated with REC-2006 alone as compared to the untreated control. However, REC-2006 treatment 2 h before irradiation decreased the expression of p53 as compared with the irradiated-only group of the HepG2 cell line, indicating the role of p53 in radiation protection. Treatment of *γ*-radiation produced more ATM cleavage product (240-kDa fragment) in the Hep3B cell line as compared with the HepG2 cell line. Inhibition of ATM cleavage as observed in HepG2 cells in a REC-2006 + radiation-treated group, suggested the role of ATM in REC-2006-mediated activation of p53 leading to radiation protection, however ATM may have a different role in the p53-deficient Hep3B cell line. Role of ATM protein in radiation protection can also be corroborated by the earlier finding, which demonstrates that AT cells are hypersensitive to DNA damaging agents, such as *γ*-radiation and restriction enzyme digestion that can introduce double-stranded DNA breaks [33]. ATM is also responsible for signaling p35-mediated cell-cycle arrest. We earlier demonstrated the REC-2006-mediated G1 phase arrest in the p53 carrying HepG2 cell line leading to higher radiation protection [2].

Increase in the expression of cytoplasmic PARP-1 in HepG2 and Hep3B cells as compared with the respective untreated control indicated the induction of PARP-1 by REC-2006. Intact (116 kDa) to cleaved (89 kDa) PARP-1 ratio was much higher in nuclear fraction of HepG2 cells as compared with Hep3B cells (∼0.91) in the radiation (−2 h REC-2006) treatment group indicating that REC-2006 inhibits the nuclear translocation of cleaved (89-kDa) PARP-1 in HepG2 cells. Furthermore, REC-2006-mediated induction of cytoplasmic PARP-1 might have inhibited the ATM kinase activity in response to DNA damage. Earlier findings that demonstrate the modulation of the ATM signaling network by a novel poly(ADP-ribose)-dependent pathway [34] and poly(ADP-ribose)polymerase-1-mediated inhibition of ATM kinase activity in response to DNA damage [35] support the present finding. Again ATM and PARP-1 participate in distinct forms of DNA repair that partially compensate for each other. PARP-1 and ATM participate in base excision repair (BER) and homologous recombination (HR), respectively [14]. *In vitro*, cell culture and *ex vivo* studies show that poly(ADP-ribosyl)ation plays a critical role in the survival and maintenance of genomic stability of proliferating cells exposed to low or moderate levels of DNA-damaging agents [16].

Expression of cytoplasmic AIF was strongly inhibited in HepG2 (p53^++^) cells pretreated with REC-2006 2 h before irradiation as compared to the irradiated control, suggesting the REC-2006-mediated inhibition of apoptosis in the HepG2 cell line. Increase in the expression of HSP in REC-2006 + radiation-treated group of the HepG2 cell line [2] supports the present finding. Ravagnan and his co-workers have already demonstrated the antagonistic relation between Hsp and AIF [36]. Similarly, the expression of nuclear AIF was found to be inhibited more in the HepG2 cell line as compared with the Hep3B cell line in the REC-2006 + radiation-treated group indicated that induction of Hsp70 in REC-2006 + radiation-treated group of the HepG2 cell line might have inhibited the nuclear translocation of AIF leading to inhibition of apoptosis. Inhibition of nuclear import of AIF to avoid DNA fragmentation in TF-1 cell has been reported earlier [37].

However, higher inhibition of cytochrome *c* and AIF as observed in the HepG2 cells and the Hep3B cells upon REC-2006 treatment 2 h prior irradiation suggests that REC-2006 inhibited the mitochondrial release of cytochrome *c* and AIF in the HepG2 cell line. The loss of Δ*ψ*_m_ accompanies the release of cytochrome *c* and AIF into the cytosol, which respectively triggers caspase-dependent and independent DNA fragmentation [37]. *Podophyllum hexandrum* mediated stabilization of mitochondrial membrane stabilization has been reported earlier [29]. Similarly, higher inhibition of Apaf-1 and caspase-9 expression observed in HepG2 cells in REC-2006 + radiation-treated group indicated that REC-2006 might have inhibited the caspase-9 by inhibiting the mitochondria release of cytochrome *c* and induction of Apaf-1. Cytochrome *c* has been shown to promote caspase-9 activation by inducing nucleotide binding to Apaf-1 [38]. Inhibition of caspase-3 observed in HepG2 cells as compared with Hep3B cells upon REC-2006 treatment 2 h prior irradiation supported the inhibition of caspase-9. Furthermore, inhibition of caspase-3 in the REC-2006 + radiation-treated group of the HepG2 cell line also supports the REC-2006 mediated inhibition of ATM and PARP-1 cleavage in the respective treatment group.

The expression of cytoplasmic ICAD was inhibited in the radiation-treated group as compared to untreated control in HepG2, as well as Hep3B cells. However, REC-2006 treatment 2 h prior to irradiation significantly restored the expression of ICAD in HepG2 cells. Inhibited expression of cytoplasmic as well as nuclear CAD observed in HepG2 cells treated with REC-2006 (−2 h) + radiation supports the restoration of ICAD in the HepG2 cell line. No REC-2006-mediated restoration of ICAD as observed in the Hep3B cell line explains the higher expression of CAD in the Hep3B cell line leading to apoptotic cell death. Restoration of ICAD in the HepG2 cell line further supports the lower nuclear to cytoplasmic CAD ratio in HepG2 cells as observed in the REC-2006 (−2 h) + irradiation treatment group of the HepG2 cell line. Lower expression of nuclear CAD and restoration of cytoplasmic ICAD explain the lower degradation of nuclear DNA as observed earlier in the HepG2 cell line.

**Figure 6:**
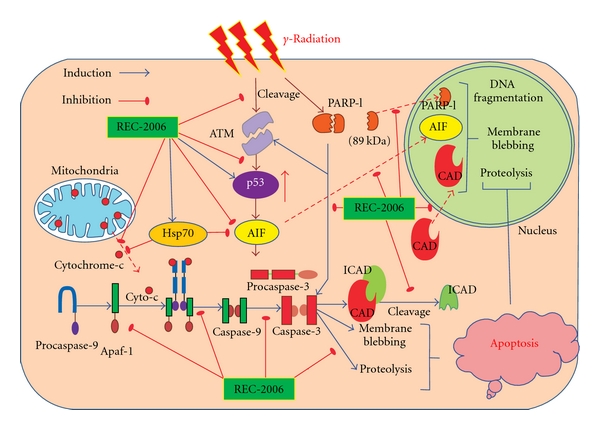
The schematic hypothetical diagram explains the *γ*-radiation-induced apoptotic and the REC-2006-mediated radiation protection pathway.

In conclusion, REC-2006 renders higher protection to the HepG2 cell line by inhibiting the expression of p53 and AIF, inhibiting the cleavage of ATM and PARP-1, and also the release of cytochrome *c* and thus modulating the expression of Apaf-1 and caspase-9 leading to inhibition of caspase-3 activities. Furthermore, REC-2006 treatment restores the expression of ICAD and inhibits the nuclear translocation of CAD and AIF leading to inhibition of DNA degradation and apoptosis. The absence of REC-2006-mediated restoration of ICAD and lower inhibition of mitochondrial release of cytochrome *c* might have induced DNA fragmentation and apoptosis that ultimately led to lower radiation protection in the Hep3B cell line.  Figure 6 explains the *γ*-radiation induced apoptotic pathways and the role of REC-2006 in modulation of apoptotic proteins lead to radioprotection. Further studies for inhibiting and activating the expression of p53 in the HepG2 cell line and transfecting the p53 gene in the p53 negative Hep3B cell line are intended to be carried out in future to validate the present observations.
